# Evaluation of Methicillin-Resistant *Staphylococcus aureus* Carriage and High Livestock Production Areas in North Carolina through Active Case Finding at a Tertiary Care Hospital

**DOI:** 10.3390/ijerph16183418

**Published:** 2019-09-14

**Authors:** Beth J. Feingold, Kerri L. Augustino, Frank C. Curriero, Paras C. Udani, Keith M. Ramsey

**Affiliations:** 1 Department of Environmental Health Sciences, Johns Hopkins Bloomberg School of Public Health, Baltimore, MD 21205, USA; 2 Department of Infection Control, Vidant Medical Center, Greenville, NC 27834, USA; kerri0718@gmail.com (K.L.A.); KRamsey@vidanthealth.com (K.M.R.); 3 Division of Infectious Diseases, Department of Internal Medicine, The Brody School of Medicine at East Carolina University, Greenville, NC 27834, USA; PudaniID@aol.com; 4 Department of Epidemiology, Johns Hopkins Bloomberg School of Public Health, Baltimore, MD 21205, USA; fcurriero@jhu.edu

**Keywords:** MRSA, MLST, rep-PCR, North Carolina, livestock, cluster detection

## Abstract

Recent reports from the Netherlands document the emergence of novel multilocus sequence typing (MLST) types (e.g., ST-398) of methicillin-resistant *Staphylococcus aureus* (MRSA) in livestock, particularly swine. In Eastern North Carolina (NC), one of the densest pig farming areas in the United States, as many as 14% of MRSA isolates from active case finding in our medical center have no matches in a repetitive sequence-based polymerase chain reaction (rep-PCR) library. The current study was designed to determine if these non-matched MRSA (NM-MRSA) were geographically associated with exposure to pig farming in Eastern NC. While residential proximity to farm waste lagoons lacked association with NM-MRSA in a logistic regression model, a spatial cluster was identified in the county with highest pig density. Using MLST, we found a heterogeneous distribution of strain types comprising the NM-MRSA isolates from the most pig dense regions, including ST-5 and ST-398. Our study raises the warning that patients in Eastern NC harbor livestock associated MRSA strains are not easily identifiable by rep-PCR. Future MRSA studies in livestock dense areas in the U.S. should investigate further the role of pig–human interactions.

## 1. Introduction

Estimates indicate that 33% of the U.S. population are colonized with *Staphylococcus aureus* (*S. aureus*), while two percent carry methicillin-resistant *S. aureus* (MRSA) [1]. MRSA colonization can lead to adverse health effects, including skin, respiratory and blood infections, as well as death [1]. In addition to hospital-associated (HA-MRSA) and community-associated (CA-MRSA) strains often seen in the U.S., livestock-associated (LA-MRSA) strains previously observed in other countries [2,3] have recently been documented in the United States [4].

The LA-MRSA sequence type-398 (ST-398) was first reported as non-typable MRSA (NT-MRSA) by pulsed-field gel electrophoresis (PFGE) in the Netherlands in 2003 [5], and is often, though not always, associated with livestock contact and living in livestock-dense areas [4]. Similar to other leading hog producing states, ST-398 has been reported among hog production workers in North Carolina (NC) [6,7], but has otherwise not previously been reported in the state.

Vidant Medical Center (VMC) is a tertiary care hospital serving the pig-dense Eastern coastal plain of North Carolina. In 2007–2009, we performed a pilot MRSA screening program using rep-PCR. In that pilot study, we found an admission prevalence of nasal carriage of MRSA of 4.2% (*n* = 390). Among the 390 MRSA isolates, 354 of these isolates had genotype data, as some positive PCR swabs did not result in a corresponding growth in culture on CromAgar. We found that 297 MRSA positives were typed by Diversilab to known strains: USA 100 (*n* = 153, 44.6%), USA 300 (*n* = 89, 26.0%), USA 200 (*n* = 11), USA 500 (*n* = 13), USA 600 (*n* = 2), USA 800 (*n* = 26), USA 1000 (*n* = 1), and Iberian (*n* = 2) [8,9]. The third largest group (*n* = 47, 13.7%) lacked a match to the rep-PCR library non-matched MRSA (NM-MRSA), raising the question of the possible presence of LA-MRSA in this livestock dense region. In the present study, we leverage this dataset to explore whether NM-MRSA strains echo the emergence of LA-MRSA in the Netherlands [5,10] by (a) investigating spatial relationships to pig density and (b) employing multilocus sequence typing (MLST) to determine if LA-MRSA strains, including ST-398, comprise the NM-MRSA group.

## 2. Materials and Methods

As previously described [8], all patients admitted to VMC (previously Pitt County Memorial Hospital) in February 2007, 2008, and 2009, were screened for nasal carriage of MRSA with the GeneOhm MRSA PCR (Becton Dickinson, San Diego, CA, USA). DNA was extracted from MRSA-positive isolates using the Mo Bio UltraClean microbial DNA isolation kit (Mo Bio Laboratories, Inc., Carlsbad, CA, USA), and molecular genotyping was performed using Diversilab (DL)™ Staphylococcus kit (bioMérieux, Inc. Durham, NC, USA) [8,11]. Diversilab strain relatedness for classification was defined as >97% similarity with no band differences, when compared to the DL reference library. PFGE types were inferred if matched to one of the strains in the DL library. Isolates with no library match were identified as a non-match (NM-MRSA). Address, age, race, sex, concurrent MRSA infection, and co-morbidities were ascertained from patient charts.

Data on numbers of pigs per county were downloaded from the United States Department of Agriculture 2007 United States Census of Agriculture (https://agcensus.usda.gov/) and land area per county was downloaded from the 2010 Tiger/Line Files (https://www.census.gov/geo/maps-data/data/tiger-line.html). Pig density per square mile per county was calculated from these data.

No official database of animal feeding operations in NC exists that is publicly available, so swine waste lagoon data (2003) was downloaded from NCOneMap (http://www.nconemap.com/) as a proxy for hog farms locations, as on-site lagoons are used for waste management in the state.

Patients’ residential addresses and hospital discharge data were geocoded and joined to counties using ArcGIS 9.3 (ESRI, Redlands, CA, USA). MRSA carriage rates per county per February were calculated as number of MRSA positives per 100 persons discharged per county per February.

A Poisson model of case counts per county, adjusting for 2010 U.S. Census county populations (https://factfinder.census.gov/), was employed to detect spatial clusters of NM-MRSA using SaTScan^TM^ cluster detection software [12] as was employed in our previous study [10]. Briefly, clusters were identified based on a likelihood ratio test comparing rates within and outside of potential clusters, with up to 10% of the population being scanned to identify clusters. Statistical significance was determined using Monte Carlo Methods [12]. Building upon the initial SaTScan model, we further controlled for county pig density to explore its influence on cluster identification in a second cluster detection model [10]. County-level data was used for this analysis because counties are the smallest administrative level for which the U.S. Agricultural Census reports on livestock. We mapped SaTScan cluster analysis results in ArcGIS.

To test whether residential proximity to hog farms was associated with NM-MRSA, we ran univariate and multivariate (adjusted for patients’ age and sex) logistic regression models in Stata (Stata Corp, College Station, TX, USA). We measured residential proximity to farms in two ways: Linear distance (as the crow flies) to closest swine lagoon and pig density per county. Multilevel logistic regression models including a county-level random intercept were tested and deemed unnecessary owing to extremely low variance of the random effect.

We performed MLST analysis (based on seven housekeeping genes: arcC, aroE, glpF, gmk, pta, tpi, and yqiL) on thawed NM-MRSA isolates from patients living in the most pig-dense areas (*n* = 12) following methods outlined in [10]. We reviewed case records for these isolates for information on previous MRSA carriage or infection (ever/within three months/never), recent hospitalization (yes/no), exposure to livestock (yes/no), and occupation to identify if any of these relevant risk factors were present.

This study was approved by the Institutional Review Board at East Carolina University/Vidant Medical Center (ECU/VMC; UMCIRB 09-0543).

## 3. Results

**Figure 1:**
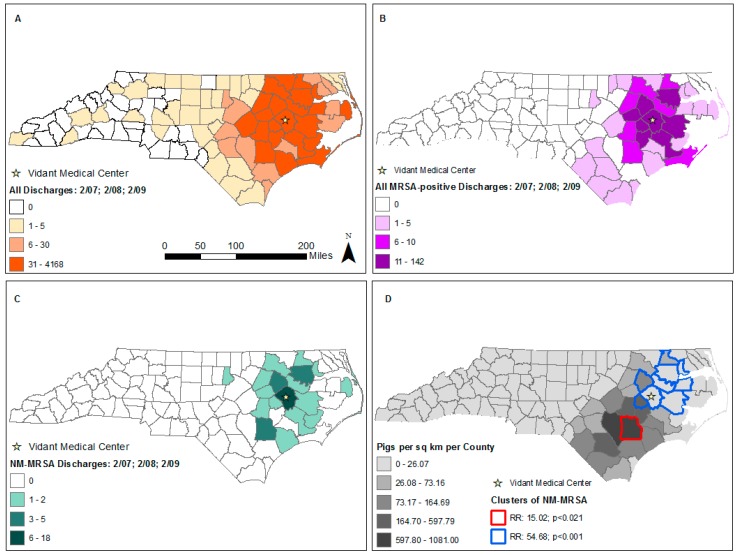
Distribution of methicillin-resistant *Staphylococcus aureus* (MRSA) isolates in North Carolina (NC) counties from active case findings at Vidant Medical Center (VMC) in February of 2007, 2008, and 2009. (**A**) All patient discharges by county. (**B**) All MRSA positives by county. (**C**) Non-matched rep-PCR MRSA positives by county. (**D**) RR (relative risk) of NM-MRSA compared to typable-MRSA from SaTScan Poisson Cluster detection analysis. These results are underlaid with pig density from 2007.

VMC discharged 2927, 3241, and 3175 patients during February of 2007, 2008 and 2009, respectively, mapped in Figure 1a. Three hundred and forty four of these 354 patients with genotyped data from the pilot study resided within the state of North Carolina (some VMC patients came from out of state) (Figure 1b). In this final dataset of MRSA-positives, 55.8% of patients were female (*n* = 192). The mean age was 56.6 years (range: 0–94). We mapped the number of NM-MRSA isolates in this dataset by county in Figure 1c.

As shown in Figure 1d, after adjusting for population size, we identified two statistically significant spatial clusters of NM-MRSA, one proximal to Vidant Medical Center and one in Duplin County, the most pig dense county (2.25 million pigs in 2007) in the state. Additional adjustment of pig density by county in a subsequent SaTScan analysis revealed no statistically significant clusters, suggesting the relevancy of pig density in explaining the spatial clusters [10].

A retrospective case–case analysis did not find higher odds of NM-MRSA compared to library matched-MRSA with regard to proximity to the nearest swine lagoon (odds ratio (OR): 1.02; 95% CI: 0.98–1.06; *p* = 0.38), nor number of pigs per county (OR: 1.05; 95% CI: 0.99–1.12; *p* = 0.09). Both were invariant to adjustments for age and sex in multivariate models.

Eleven of the 12 samples that we sent for MLST analysis were detectable and identified as follows: ST-398 (*n* = 1), ST-5 (*n* = 3), ST-840 (*n* = 2), community-associated (CA-MRSA) strains ST-72 (*n* = 3) and ST-8 (*n* = 1), and the hospital associated (HA-MRSA) strain ST-36 (*n* = 1).

**Figure 2:**
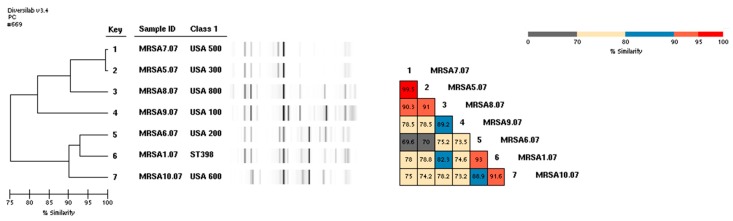
Rep-PCR generated dendrograms for six MRSA reference strains (#1–5, 7) and one MRSA ST-398 (#6). The gel-like image generated by the Diversilab software illustrates band similarities.

Figure 2 shows the dendrogram of known MRSA USA types [9], and the MLST-identified MRSA ST-398 isolate with its distinct banding pattern. In addition, the three ST-5 isolate fingerprints showed high similarity. Two showed 99.1% similarity, while a third was near 91% similarity (data not shown).

## 4. Discussion

ST-398 are an emergent MLST type associated with livestock, particularly swine. In this study, we found that MRSA isolates belonged to ST-5 and ST-398 among patients at our medical center, confirming their presence in North Carolina [7,11]. Importantly, these types represented rep-PCR patterns that were heretofore uncharacterized in the U.S.

Since not all ST-398 are livestock associated, we lack evidence to conclude with certainty that the one ST-398 isolate identified is a livestock associated isolate. While the medical record indicates that the ST-398 isolate belonged to a retired farmer in a pig-dense area, we lack detailed patient information regarding farm type and whether there was any livestock exposure at the time of hospital admission. We tested VMC’s internal library (>2500 MRSA isolates) from 2009–present, including those previously evaluated in our studies [8,11] and found zero additional isolates with the same distinct rep-PCR pattern as the ST-398 identified in this study; thus, among VMC’s patient populations, ST-398 carriage is rare. None of the other tested MLST isolates were known to be from persons associated with farming. The three isolates of ST-5 belonged to a housewife, a salesperson and an adult with an unknown occupation, respectively. The third of these persons had been disabled after multiple cardiac illnesses.

The banding pattern of our MRSA MST-398 isolate, identified in the Diversilab library as non-typable, differs somewhat from previously published patterns from Austria [13]; these data suggest that there is some heterogeneity among MRSA ST-398 banding patterns using the rep-PCR typing methods.

While MRSA carriage in our population is approximately double the CDC’s estimates of percentage of carriage in the general population [1], it is comparable to the 4.0% detected via active surveillance at nearby Medical University of South Carolina [14]. This higher percentage likely represents a risk of MRSA carriage among those seeking hospital care versus the general population.

The results of the MLST testing on 25% of our NM-MRSA isolates indicate heterogeneity of hospital-associated and community-associated strains within the NM-MRSA group. This heterogeneity differs from the homogeneity observed previously among the PFGE non-typable MRSA (NT-MRSA) isolates from the Netherlands [5]. The percentage of MRSA USA 300 in the present study was identical to that of the Medical University of South Carolina’s study referenced previously, suggesting similar rates of carriage in this geographic area [14].

This study is limited in several ways. First, it is possible that we have misclassified exposure to farms due to our use of proxy measures of exposure (e.g., distance to farm), the slight temporal discordance between the swine lagoons dataset and patient records, and our inability to account for secondary transmission and all relevant occupational exposures. Second, we lacked resources to conduct MLST on all 47 non-matched isolates. Lastly, spatial cluster detection analysis is always subject to ecological bias and the modifiable area unit problem (e.g., the arbitrary boundaries of counties), which may have biased cluster detection results.

Finally, though the number of pigs per county was not a statistically significant variable in the logistic regression model, the addition of pig density per county had a statistically significant impact on our spatial cluster detection analysis. This discrepancy may be due to an unmeasured confounder or the differing analytic strategies between the two types of models.

## 5. Conclusions

This study confirms presence of MRSA ST-5 and ST-398 in a pig-dense region of North Carolina. This work contributes to the understanding of human colonization with epidemiologically important pathogens in the U.S. It adds to the growing body of literature underscoring the need for MRSA surveillance, including strain typing, in U.S. hospital systems located within and outside of agricultural communities, in order to better understand the relationships between livestock-associated MRSA and humans [15]. More extensive studies are needed in the U.S. on the pig–human connection to delineate its role in the emergence of novel MRSA strains to inform new methods of control.
